# Gender differences in the impact of short video addiction on depression among college students: the mediating effect of self-concept clarity

**DOI:** 10.3389/fpsyg.2026.1771454

**Published:** 2026-03-30

**Authors:** Dongning Zhang, Jing Wu, Jing Li, Yifu Yang, Yuan Xing

**Affiliations:** School of Public Health, Xi’an Medical University, Xi’an, China

**Keywords:** depression, gender differences, mediating effect, self-concept clarity, short video addiction

## Abstract

**Background:**

The negative impact of short video addiction (SVA) on the mental health of college students is a current global public health issue. This research explored the mediating role of self-concept clarity (SCC) in the relationship between SVA and depression among college students.

**Methods:**

A questionnaire survey was administered to 510 college students using the Short Video Overuse Behavior Scale, the Self-Concept Clarity Scale, and the Center for Epidemiologic Studies Depression Scale. Using AMOS 26.0 to establish an SEM and test the mediating effect of SCC, and multi-group analysis was used to examine gender differences in mediating effect.

**Results:**

A positive association was observed between SVA and depression, however, further analysis revealed no significant direct effect. Instead, the relationship between SVA and depression was fully mediated by SCC, which exhibited negative associations with both variables. Moreover, a significant gender difference emerged, with this indirect pathway being significant only among female college students.

**Conclusion:**

SVA may be indirectly associated with depressive symptoms among college students through the mediating effect of SCC, and this mediating effect was significant only among female students.

## 1 Introduction

Short videos refer to online video clips with a duration ranging from seconds to minutes. They are primarily distributed through social media, video-sharing websites, and mobile apps (Dong et al., 2023). On short video platforms, users can watch, create, share videos and live streams, as well as interact through comments, enabling them to fulfill their leisure, social, and informational needs. Simultaneously, short video platforms recommend videos to viewers based on users’ personalized needs, allowing users to derive tremendous psychological gratification. The consumption of short videos is especially popular among young people, among whom college students comprise the main user group because they have relatively more time for leisure and a strong sense of curiosity. A survey showed that the proportion of Chinese college students who watch short videos for more than 2 hours a day ranged from 26.45–36.1% (Xie and Jia, 2021; Ai et al., 2023).

### 1.1 Short video addiction and depression

The popularity of short video platforms has brought great convenience to people but has also brought about the problem of overuse or addiction to short videos. Similar to Internet addiction, SVA is characterized by a compulsive inability to control short video usage, which leads to significant negative consequences for individuals (Zhang et al., 2019). SVA is a branch of Internet addiction, and many studies have considered it a manifestation of social media addiction (Appel et al., 2018; Masciantonio et al., 2021; Karakose et al., 2023). A survey found that 21.6% of Chinese college students have SVA symptoms (Li et al., 2021). Moreover, SVA is associated with interpersonal problems (Huang et al., 2021), lower subjective well-being (Ye et al., 2022), and depressive symptoms (Liang et al., 2020; Chao et al., 2023; Qu et al., 2024; Zhu et al., 2024). These associations are particularly pronounced among young individuals with a higher susceptibility to depression (Yao et al., 2022).

According to the social displacement hypothesis (Kraut et al., 1998), overusing the Internet causes people to consume a great amount of time and energy, which leads to fewer interactions between them and their family and friends while also reducing their sense of security and belonging, thereby resulting in depression. However, research has found a complex link between social media usage behaviors and depression. One line of research focuses on general social media use (e.g., duration and frequency of use), and findings in this area are currently controversial. For example, the American Academy of Pediatrics has published a report pointing out that using social media such as Facebook may contribute to depression in children and adolescents (O’Keeffe et al., 2011). However, some studies have indicated that social media use is not a strong or consistent risk factor for depressive symptoms (Kreski et al., 2021). Furthermore, several studies have found no direct association between social media use and depression. Rather, mediating mechanisms—such as jealousy and social comparison—play a crucial role in this relationship (Jelenchick et al., 2013; Tandoc et al., 2015; Ding et al., 2016; Tian et al., 2020). Another line of research focuses on problematic or addictive social media use. Regarding the relationship between this pattern of use and depression, existing studies have reached relatively consistent conclusions, indicating that addictive use is positively correlated with depressive symptoms (Liang et al., 2020; Qu et al., 2024). The variable of interest in this study is “SVA,” which falls within the category of problematic use. Although this main effect relationship has been confirmed in multiple studies, the underlying psychological mechanism—namely, “how SVA is associated with depression”—still requires in-depth exploration. Therefore, this study introduces self-concept clarity as a mediating variable, aiming to reveal the internal mechanism of this influence process.

### 1.2 Mediating effect of SCC

According to the cognitive theory of depression, individuals negatively perceiving themselves, the external world and their future is the major cause of depression. Research shows that low SCC is an important factor of depression (Lee-Flynn et al., 2011; Ding et al., 2016; Yang et al., 2017). SCC refers to one’s lucid, confident, and clear understanding of themselves while possessing the qualities of internal consistency and stability at the current stage (Campbell et al., 1996). Higher SCC can facilitate positive emotions, such as well-being (Nie and Gan, 2017) and strengthen the ability to cope with conflict (Smith et al., 1996; Zhou et al., 2012), whereas lower SCC is associated with the emergence of negative emotions (Bigler et al., 2001; Yang et al., 2017), which may impair the development of the affected individual (Krol et al., 2019; Parise et al., 2019; Alessandri et al., 2021). Research has found that Internet addiction is associated with lower SCC (Appel et al., 2018; Petre, 2021). According to the self-concept fragmentation hypothesis (Valkenburg and Peter, 2011) the Internet provides various potential interactive partners with ways for people to try out their identities. Users exposed to diverse roles and values, especially teenagers, find it difficult to integrate all choices into a coherent and unified sense of self, thereby increasing the risk of personality fragmentation and self-concept confusion. Compared to traditional social media platforms (such as Facebook and WeChat), short video applications have unique media characteristics that may exhibit a more pronounced association with SCC (Smith and Short, 2022; Zhang et al., 2023). First, short video platforms employ algorithm-driven recommendation mechanisms that continuously push highly personalized and fragmented content to users (Montag et al., 2021). This mode of content presentation may contribute to a fragmented self-perception. Second, short video platforms prioritize entertainment over interpersonal interaction (Chao et al., 2023), placing users in a state of passively receiving information rather than actively constructing stable social networks. Third, short videos are replete with carefully curated and idealized portrayals of others’ lives (Su et al., 2021), providing abundant material for upward social comparison. The combined effect of these features may make it more difficult for users—particularly college students who are in a critical period of identity formation—to maintain a clear and stable self-concept. Therefore, we hypothesize that SVA is negatively associated with SCC, and that SCC mediates the relationship between SVA and depression.

### 1.3 Gender differences in SVA

Research has shown significant gender differences in Internet addiction and its consequences (Liang et al., 2016). Although males are more prone to internet overuse than females (Scherer, 1997; Li et al., 2014), the detrimental effect of Internet addiction on women is more serious than that on men (Ko et al., 2014; Li et al., 2014; Liang et al., 2016; Park and Lee, 2022; Yen et al., 2009). This phenomenon is particularly evident in social media use.

From the perspective of social comparison theory, female users are more inclined to focus on others’ evaluations and engage in social comparison during the socialization process (Nesi and Prinstein, 2015). The contrast effect generated by upward social comparison is associated with lower self-evaluation levels (Collins, 1996), increase dissatisfaction with body image (Arroyo, 2014), and be associated with reduced SCC (Butzer and Kuiper, 2006; Niu et al., 2016). From the perspective of self-presentation, females are more willing to actively present themselves on social networks and pay greater attention to others’ positive images on these platforms (Eckler et al., 2017). This heightened tendency toward self-presentation and social comparison may make females more susceptible to the negative effects of social media. From a developmental psychology perspective, college students are in a critical period of identity formation (Wang and Chen, 2013), and female college students show significantly greater concern about appearance than their male counterparts (Bu and Gao, 2021; Carter and Vartanian, 2022). Appearance anxiety, as an important component of self-concept, is more prominent among females (Zhu and Yin, 2023). When female college students are exposed to numerous meticulously curated appearance images on short video platforms, they are more likely to doubt their own appearance through social comparison, which may be associated with reduced SCC and negative emotions (Tian et al., 2020).

Based on the above analysis, we hypothesize that the effects of SVA on SCC and depression may exhibit significant gender differences, with females being more vulnerable to negative impacts.

### 1.4 The present study

College students are in a transitional phase from adolescence to young adulthood. Therefore, they inevitably have to face a lot of difficulties and stressors in their daily life, which makes them a group of individuals with high risk for depression (You et al., 2016). College students are also in a crucial stage of forming their ego identity (Wang and Chen, 2013) and are easily disturbed by negative online content, leading to confusion in their self-concept. In addition, college students are the chief group of short video users; As such, we focused on college students to explore the mediating effect of SCC between SVA and depression, and we formulated the following hypotheses.

*Hypothesis 1*: SVA can positively and directly predict depression among college students.

*Hypothesis 2*: SVA be associated with depression through the mediating effect of SCC.

*Hypothesis 3*: There are gender differences in both the direct effect of SVA on depression and its mediating effect through SCC.

## 2 Materials and methods

### 2.1 Participants and procedures

This study was conducted through an online survey in December 2023, which recruited 613 college students from two universities in Xi’an, Shaanxi Province, China. The questionnaire included information on demographic traits, short video usage habits, SVA, SCC and depression. Excluding participants who filled out attention detection questions incorrectly, and participants who answered ‘never watch short videos’, a total of 510 valid responses were obtained, with an effective rate of 83.20%. Among them, there were 136 males, accounting for 26.67%, and 374 females, accounting for73.33%. The average age and standard deviation of the participants were 20.22 and 1.34, respectively. The studies were approved by the Medical Ethics Review Committee of Xi’an Medical University. All participants provided written informed consent and retained the right to withdraw from the study at any time.

The proportion of female participants in this study’s sample was significantly higher than that of male participants. This ratio reflects, to some extent, the gender distribution characteristics of the two universities, but it may also have potential implications for the analysis of gender differences. When conducting gender difference analyses, we employed more stringent statistical criteria (e.g., critical ratios for differences between parameters) and fully consider potential power issues arising from sample imbalance in the interpretation of results.

### 2.2 Measures

#### 2.2.1 Questionnaire on short video usage habits

We compiled a questionnaire on short video usage habits that included five items:

- “Please estimate your average daily time spent viewing short videos.” The options were *never watching*, *within 1 h*, *1–2 h*, *2–3 h*, and *over 3 h*.
- “Do you leave comments while watching short videos?”
- “Do you have the habit of shooting and posting short videos?”
- “Would you like to appear in your own posted short videos?”
- “Would you forward or share short videos with acquaintances?”

Items 2 to 5 used a 4-point rating system: 1 = *never*, 2 = *occasionally*, 3 = *frequently*, and 4 = *extensively*.

In this study, the short video usage habits questionnaire was primarily used to describe the basic usage characteristics of the sample and to provide behavioral-level background information for subsequent gender difference analyses. These variables were not included in the main structural equation modeling analysis but served as supplementary evidence when interpreting gender differences.

#### 2.2.2 Short video overuse behavior scale

This scale was formulated by Wang et al. (2020). It contains seven items, such as:

- “Watching short videos on my phone takes up a lot of my time.”
- “Whenever I have free time, I watch short videos on my phone.”
- “I will frequently open my phone to watch the latest short videos”

The answers are rated using a 5-point rating system (1 = *completely inconsistent* and 5 = *completely consistent*). Higher total scores indicate the existence of more severe SVA. The Cronbach’s *α* was 0.89 in this study. The confirmatory factor analysis yielded satisfactory results across all measurements. χ^2^/df = 1.657, TLI = 0.992, CFI = 0.996, GFI = 0.991, RMSEA = 0.036, RMR = 0.019.

#### 2.2.3 Self-Concept Clarity Scale

This scale was formulated by Campbell et al. (1996) and revised by Niu et al. (2016), which is consistes of 12 items, such as:

- “My opinions about myself often conflict with those that other people have about me.”
- “Regarding myself, I have this idea today, and tomorrow I will have another idea.”
- “I spend a lot of time thinking about what kind of person I really am.”

The answers are rated using a 5-point rating system (1 = *completely inconsistent* and 5 = *completely consistent*). Except for items 6 and 11, all other items were rated in reverse. Higher scores correspond to higher degrees of SCC. The Cronbach’s *α* was 0.81 in this study. The model fit indices for the confirmatory factor analysis indicated a favorable fit across all measurements. χ^2^/df = 2.414, TLI = 0.932, CFI = 0.955, GFI = 0.966, RMSEA = 0.053, RMR = 0.039.

#### 2.2.4 Center for Epidemiologic Studies Depression Scale (CES-D)

This scale was formulated by Radloff (1977) and further revised by Zhang et al. (2010); it encompasses 20 distinct items, such as:

- “I feel that even with the help of friends, I cannot escape this sense of frustration”
- “I think there is still hope for the future”
- “I do not want to eat anything. I have a bad appetite”

The participants responded using a 4-point rating system (1 = *rarely or none of the time*, 2 = *some or a little of the time*, 3 = *occasionally or some of the time*, and 4 = *all of the time*) and had to answer based on whether their circumstances during the past week matched the items. Higher total scores indicate the existence of more severe depression. The Cronbach’s α was 0.92 in our study. The confirmatory factor analysis demonstrated adequate model fit for all measures. χ^2^/df = 1.531, TLI = 0.981, CFI = 0.985, GFI = 0.958, RMSEA = 0.032, RMR = 0.030.

### 2.3 Data analysis

The data analysis was conducted using SPSS 25.0, which included reliability analysis, descriptive statistics, a chi-square test, an independent-samples t-test, correlation analysis, and an assessment of common method bias (CMB). Structural equation modeling (SEM) and multi-group analysis were conducted utilizing AMOS 26.0. The significance of the mediating effect was assessed utilizing a bootstrapping method with 2,000 samples. The results revealed that the 95% bias-corrected confidence intervals (CIs) did not include zero, confirming a statistically significant effect. The model’s goodness of fit was evaluated using the following criteria: χ^2^/df < 3, CFI > 0.90, TLI > 0.90, RMSEA < 0.08, and SRMR < 0.05. The threshold for statistical significance was set at *p* < 0 0.05.

## 3 Results

### 3.1 Common method bias test

We used Harman’s single-factor test to determine the presence of CMB. We extracted a total of 10 factors with eigenvalues greater than 1, which explained 58.8% of the variance; the explanation rate of the first factor was 25.13%, which is lower than the critical value of 40%, indicating no serious CMB in this study.

### 3.2 Short video usage habits

Table 1 shows that 77.84% of college students used short video applications for more than 1 h per day, 37.25% for more than 2 h, and 14.12% for more than 3 h. The chi-square test indicates no significant difference in short video usage time between men and women.

**Table 1:** Distribution of short video usage times and sex differences.

Time	Total (510)	Men (136)	Women (374)	χ^2^
Within 1 h	113 (22.16%)	31 (22.79%)	82 (21.93%)	2.41
1–2 h	207 (40.59%)	48 (35.29%)	159 (42.51%)	
2–3 h	118 (23.14%)	35 (25.74%)	83 (22.19%)	
Over 3 h	72 (14.12%)	22 (16.17%)	50 (13.37%)	

Table 2 suggests that there were significant gender differences in “shooting and publishing,” “self-presentation,” and “sharing and forwarding,” with women having a higher score than men. This outcome indicates that compared to men, female college students prefer to create and post short videos and also prefer to showcase themselves in short videos and share them with friends or acquaintances.

**Table 2:** Descriptive statistics and sex differences (M ± SD) (*N* = 510).

Variables	Total (510)	Men (136)	Women (374)	*t*
Commenting	1.82 ± 0.63	1.75 ± 0.66	1.84 ± 0.61	−1.47
Shooting and publishing	1.59 ± 0.65	1.46 ± 0.63	1.65 ± 0.64	−3.02**
Self-presentation	1.76 ± 0.73	1.60 ± 0.73	1.82 ± 0.72	−2.96**
Sharing and forwarding	2.49 ± 0.83	2.28 ± 0.86	2.56 ± 0.81	−3.46**
SVA	20.64 ± 5.34	21.24 ± 6.03	20.42 ± 5.05	1.41
SCC	37.99 ± 6.82	38.19 ± 7.22	37.92 ± 6.68	0.39
DEP	33.60 ± 10.52	35.10 ± 12.30	33.06 ± 9.76	1.74

**p < 0.01.

### 3.3 Descriptive statistics, gender differences, and correlation analysis

Table 2 shows no significant gender differences in SVA, SCC, or depression. Table 3 presents the correlation analysis, indicating a significant positive correlation between SVA and depression, and a significant negative correlation between SVA and SCC, as well as between SCC and depression. This result implies that the more severe the SVA, the lower the SCC and the more severe the depression.

**Table 3:** Correlations of the study variables (*N* = 510).

Variables	1	2	3	4	5
Sex	1				
Age	−0.14**	1			
SVA	−0.07	−0.00	1		
SCC	−0.02	0.07	−0.27**	1	
Depression	−0.09	−0.00	0.18**	−0.49**	1

In terms of sex, 1 = male and 2 = female; **p < 0.01.

### 3.4 Mediation effect test

We performed latent variable SEM with SVA as the independent variable, depression as the dependent variable, and SCC as the mediating variable. The SCC measures were partitioned into three parcels each, adhering to the item-to-construct balance principle (Little et al., 2002). This technique entails grouping items by factor loading magnitude, wherein the cumulative loadings are balanced across all resultant parcels. We observed depression along four dimensions: depressed affect (DA), positive affect (PA), somatic and retarded activity (SA), and interpersonal (IP). The fitting indexes of the structural equation are *χ^2^/df* = 1.688, TLI = 0.978, CFI = 0.983, RMSEA = 0.037, SRMR = 0.039. These indices suggest that the model fits the data well.

**Figure 1:**
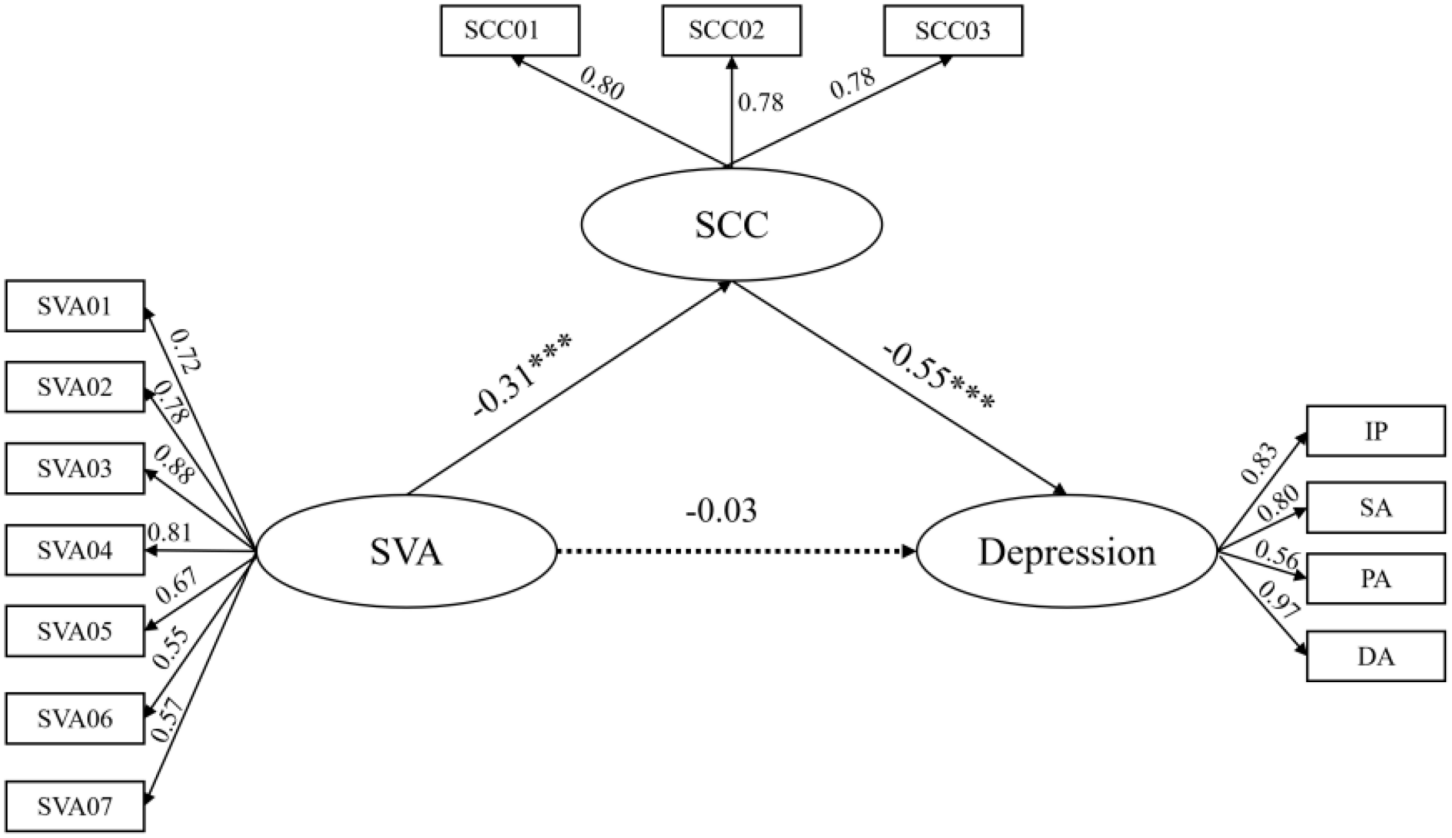
Mediating effect model of SCC between SVA and depression. DA, depressed affect; PA, positive affect; SA, somatic and retarded activity; IP, interpersonal. ***p < 0.001. In this study, the DA dimension of the CES-D exhibited an extremely high factor loading (0.97), indicating that it is a very strong indicator of the depression construct within this specific sample. Although this value is exceptionally high, the overall measurement model demonstrated good fit, and therefore we retained all dimension to preserve the integrity of the scale.

The mediation analysis results showed (see Figure 1) that the indirect effect of SVA on depression through SCC was significant (*β* = 0.17, 95% CI: [0.11, 0.25]), indicating that SCC mediates the relationship between SVA and depression. Further analysis of the direct effect revealed that, after including SCC, the direct path from SVA to depression was not significant (*β* = −0.03, 95% CI: [−0.14, 0.08]), suggesting that SCC fully mediates the relationship between SVA and depression. Specifically, SVA significantly and negatively predicted SCC (*β* = −0.31, 95% CI: [−0.43, −0.19]), and SCC significantly and negatively predicted depression (*β* = −0.55, 95% CI: [−0.62, −0.46]). Consequently, Hypothesis 1 (the direct effect of SVA on depression) was not supported, whereas Hypothesis 2 (the mediating role of SCC) was supported.

In terms of effect size, both the predictive effect of SVA on SCC (*β* = −0.31) and SCC on depression (*β* = −0.55) reached moderate levels (Cohen, 1988), indicating that SCC plays a substantial mediating role in this relationship. This means that for every one unit increase in SCC, the level of depression would be expected to decrease by 0.55 units—an effect that holds practical significance for mental health interventions.

### 3.5 Multi-group analysis by gender

To investigate whether there were gender differences in the mediating model, we established mediation effect models for men and women. The fitting indicators of the model for men are: *χ^2^/df* = 1.16, CFI = 0.99, TLI = 0.99, GFI = 0.91, RMSEA = 0.03, SRMR = 0.06. The fitting indicators for the model for women are: *χ^2^/df* = 1.65, CFI = 0.98, TLI = 0.97, GFI = 0.96, RMSEA = 0.04, SRMR = 0.04. These indicators show that both models fit well, and a comparison of their results across genders is valid.

We performed a multi-group analysis using gender as a categorical variable, and we established an unrestricted model (M1), a measurement weights model (M2), a structural weights model (M3), and a structural covariance model (M4). Table 4 indicates that the models fit the data well. The comparison results of the models suggest that the differences in fitting indices (ΔCFI, ΔRMSEA) are less than 0.01 between each model pairwise, implying that the equivalent models are valid (Cheung and Rensvold, 2002). These findings denote that the models mediated by SCC have the same meaning and potential structure for both male and female college students.

**Table 4:** Fit indices and equivalence tests of various models in multi-group analysis.

Model	χ^2^	df	CFI	TLI	GFI	RMSEA	ΔCFI	ΔRMSEA
M1	230.359	76	0.982	0.977	0.943	0.028		
M2	260.130	65	0.977	0.973	0.936	0.031	0.005	0.003
M3	268.354	60	0.977	0.973	0.934	0.031	0	0
M4	271.864	57	0.976	0.973	0.932	0.031	0.001	0

**Figure 2:**
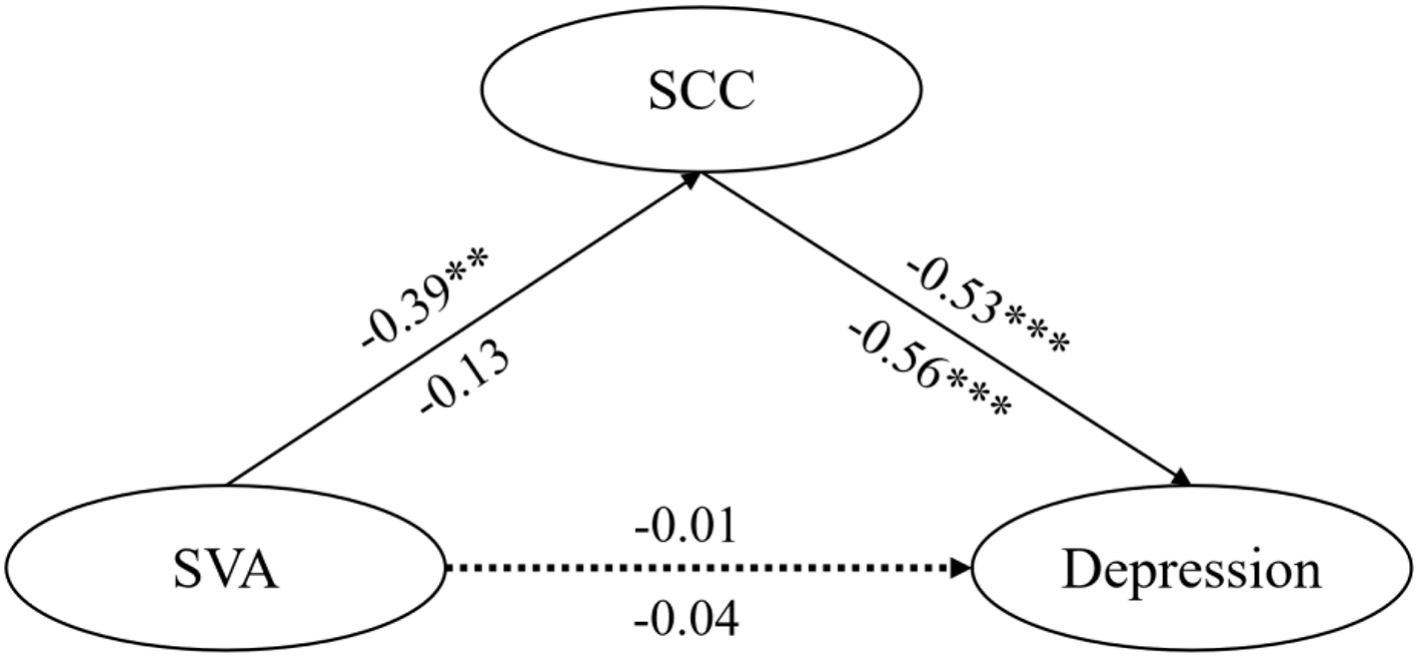
Standardized path coefficients of the mediation model in the male and female groups. The path coefficients outside the parentheses are for female students, while those inside the parentheses are for male students; ***p < 0.001.

To determine whether there was a gender difference in the path coefficient in the structural model, we performed an equivalence analysis between the unrestricted and structural weights models. According to the comparison of nested models, there was a significant difference between the two models (Δ*χ^2^* = 37.995, Δdf = 16, *p* < 0.01). According to Figure 2, after comparing the regression coefficients of different groups on the path of the structural model using the critical ratio of differences between parameters, we found no significant difference in the path coefficients from SVA to depression (*β*_male_ = −0.04, *β*_female_ = −0.01, CR = 0.347, *p* > 0.05) or from SCC to depression (*β*_male_ = −0.56, *β*_female_ = −0.53, CR = 1.49, *p* > 0.05). However, there was a significant difference in the path coefficient between SVA and SCC (*β*_male_ = −0.13, *β*_female_ = −0.39, CR = −3.16, *p* < 0.05). The above results indicate that for male college students, the direct predictive effect of SVA on SCC was not significant (*β* = −0.13, 95% CI: −0.39 – 0.09). In contrast, a significant direct effect was observed among female college students (*β* = −0.39, 95% CI: −0.51 to −0.28). Additionally, the total effect was estimated at 0.20 (95% CI: 0.04 to 0.36). The results partially support Hypothesis 3: For female college students, SVA can have a positive predictive effect on depression through the fully mediating effect of SCC.

The proportion of female participants in the study sample was substantially higher than that of male participants. While this distribution partly reflects the demographic characteristics of the partner institutions, it may nonetheless influence the interpretation of the findings, particularly those related to gender differences. To evaluate the potential impact of this limitation, we conducted a post-hoc power analysis. The results revealed that, for the male subgroup, under the condition of a two-tailed test with *α* = 0.05, the statistical power for the path coefficient from SVA to SCC was below the conventional threshold of 0.80 given the current sample size, indicating insufficient statistical power. This suggests that the effect may be genuinely weak or absent among males, but it also raises the possibility that a smaller true effect could not be detected due to the limited sample size in this group.

## 4 Discussion

### 4.1 Analysis of short video usage habits

The questionnaire survey showed that, except for a very small number of college students who did not use short video applications, over 37.25% watched short videos for more than 2 hours per day. These results are consistent with previous studies (Ai et al., 2023). There was no significant difference in the duration of short video usage between male and female college students. In terms of other usage habits, except for “commenting,” female college students scored significantly higher than male students in terms of “shooting and publishing,” “self-presentation,” and “sharing and forwarding.” These findings suggest that female college students prefer to showcase themselves in short videos and use social interaction functions in short video applications. These findings align with those of prior studies (De-Sola et al., 2016; Liang et al., 2016; Chen et al., 2017; Eckler et al., 2017).

### 4.2 Relationship between SVA and depression

The correlation analysis revealed a positive correlation between SVA and depression, indicating that the stronger the SVA, the more severe the depression. This result aligns with those of past studies (Liang et al., 2020; Yao et al., 2022; Karakose et al., 2023; Qu et al., 2024; Zhu et al., 2024). However, the mediation effect model demonstrated that SVA could not directly predict depression in college students; rather, SVA was indirectly associated with depression through the fully mediating effect of SCC. This outcome is consistent with some research findings on the usage behavior of SNS (Ding et al., 2016; Xu et al., 2017; Yang et al., 2017; Li et al., 2018). Some studies suggest that SNS usage behavior is not necessarily directly associated with depression; rather, it is the social comparison linked to SNS use (Lee, 2014), and envy or a decrease in SCC related to social comparison that are key factors in depression (Jelenchick et al., 2013; Tandoc et al., 2015; Ding et al., 2016; Tian et al., 2020). We found that although there are some differences between short video applications and traditional SNS, they have similar mechanisms in their relationship with depression. In other words, SVA may not be directly associated with depression; rather, a decrease in SCC associated with SVA appears to play a key role in depressive symptoms.

### 4.3 Mediating effect of SCC

The correlation analysis indicates that SCC was negatively correlated with both SVA and depression, implying that the stronger the SVA, the lower the SCC and the more severe the depression, which aligns with previous findings (Niu et al., 2016; Appel et al., 2018; Alessandri et al., 2021; Petre, 2021). The mediation effect model revealed that SCC had full mediation between SVA and depression. The self-concept fragmentation hypothesis denotes that individuals on the Internet can readily explore and experience the different aspects of their identity; this not only puts them at risk of not being able to integrate different aspects of themselves but also disintegrates the unified, stable self that the individual has already formed (Valkenburg and Peter, 2011). A meta-analysis indicated a significant, negative correlation between general Internet use and SCC (Petre, 2021). Longitudinal studies have demonstrated that the intensity of Facebook usage negatively predicts SCC (Appel et al., 2018). The use of SNS is associated with upward social comparisons, which may contribute to doubts, confusion, and even denial of one’s existing self-awareness (Niu et al., 2016; Liu et al., 2017; Wu et al., 2020). Short videos have not only the entertainment and information search functions of traditional online platforms but also powerful social interaction functions. Hence, when college students use short videos, they are exposed to diverse perspectives and content, which may promote upward social comparison and further confusion in their self-concept, potentially contributing to decreased SCC.

The cognitive theory of depression states that an individual’s negative cognition of themself, the world, and the future causes depression. Low self-esteem can predict depression significantly (Mor and Winquist, 2002). Similarly, SCC is positively correlated with self-esteem as a type of understanding and perception of oneself (Kawamoto, 2020). Individuals with lower SCC may struggle to establish stable self-esteem due to a confused self-perception, which is associated with negative self-evaluation and, consequently, depressive symptoms (Wong et al., 2016).

It is important to note that while our model supports a full mediation effect, wherein the direct effect of SVA on depression was non-significant after accounting for SCC, this finding should be interpreted with caution. The conclusion of full mediation is contingent upon the variables included in the current model. It is plausible that other unmeasured mediators, such as sleep quality (Hu et al., 2021), fear of missing out (FoMO), or real-world social support, may also play a role in the relationship between SVA and depression. Therefore, our finding that SCC serves as a primary and dominant mediator highlights its crucial importance, but does not preclude the existence of other explanatory pathways.

### 4.4 Gender differences in the mediation effect model

The results showed no gender differences between male and female college students in terms of short video usage time, SVA, SCC, or depression, but the multi-group analysis uncovered a significant gender difference in the relationship between SVA and SCC in the mediation effect model. Specifically, for female college students, SVA had a negative predictive effect on SCC; however, for male students, this effect was insignificant.

Research on Internet and mobile phone addiction indicates that men are more focused on the entertainment functions of the Internet while women are more focused on its social interaction functions (De-Sola et al., 2016; Liang et al., 2016; Chen et al., 2017). Research on SNS has shown that women are more willing than men to actively present themselves on social networks and pay more attention to the positive image of others on social networks. They are more willing than men to display photos of positive self-images on social networks while comparing their own image with that of others to enhance their ideal self-experience (Eckler et al., 2017). The survey results on short video usage habits also confirmed the above conclusions. College students are typically in a mature stage of adolescence and are often highly sensitive about their own appearance as well as that of their peers and the opposite gender. This situation is more prominent among female college students (Bu and Gao, 2021; Carter and Vartanian, 2022), who have significantly higher appearance anxiety (an important aspect of an individual’s self-concept) than men (Zhu and Yin, 2023). Female college students pay more attention to their own appearance, which makes them prefer to display beautiful pictures or videos online as well as pay attention to fashion and beauty content (Tian, 2023). Through social comparison, they may develop doubts about their appearance, which is associated with lower SCC, heightened negative emotions (Tian et al., 2020), and ultimately, greater depressive symptoms.

It should be noted that the sample size for male participants in this study was substantially smaller than that for female participants. Post-hoc power analysis indicated that the statistical power for the path from SVA to SCC in the male sample was below 0.80, suggesting that the observed non-significant predictive effect of SVA on SCC among males may be partly attributed to insufficient statistical power to detect a small effect size due to the limited sample size. Therefore, although this study identified a significant mediating effect in the female population, whether a weak but genuine mediating effect exists in the male population requires further validation in studies with larger sample sizes and more balanced gender ratios.

### 4.5 Implications, limitations, and future research

This study examined the relationship between SVA and depression among college students. At the theoretical level, this study reveals the fully mediating role of self-concept clarity in the relationship between SVA and depression, and finds that this mediating effect is significant only among females. This provides a new perspective for understanding the gender-specific mechanisms underlying the mental health impact of short video use. The finding extends the self-concept fragmentation hypothesis to the context of short video platforms and deepens the understanding of how addictive behaviors influence emotional outcomes through self-perception pathways. At the practical level, the study identifies enhancing self-concept clarity as an actionable target for mental health interventions. Particularly for female college students, differentiated guidance strategies may be designed to foster rational self-perception and reduce negative emotions arising from social comparison. Moreover, the findings provide a scientific basis for media literacy education, helping students critically evaluate short video content and mitigate the potential impact of algorithm-driven recommendations and idealized portrayals on self-concept. As such, the study has strong practical value.

As for the limitations and future research, first, given that this study employed a cross-sectional design, it cannot establish causal relationships among the variables. Therefore, the proposed model—in which SVA predicts depression through the mediating role of SCC—may also accommodate alternative directional pathways. For example, depression could predict SVA via SCC, or SCC might predict SVA through depression. These possibilities warrant further investigation using experimental or longitudinal designs in future research. Second, the imbalanced gender ratio in the sample and its implications for interpreting the results. Despite the issue of sample imbalance, our multi-group analysis using critical ratios for differences between parameters revealed that the effect of SVA on SCC was significantly stronger in females than in males. This cross-sample comparison provides some evidence for the robustness of the moderating effect of gender. Future studies are needed to re-examine these findings in samples with more balanced gender ratios, particularly those including more male participants, in order to clarify whether the effect of SVA on SCC in the male population is genuinely non-significant or simply reflects a small effect size. Third, this study focused exclusively on Chinese college students, and this cultural context may have exerted specific influences on the results. Chinese society is characterized by a collectivist cultural tradition, in which individuals’ self-concept formation is more susceptible to others’ evaluations and social comparisons (Niu et al., 2016). Moreover, Chinese college students face considerable academic and employment pressures, which may heighten their need for the entertainment and emotional regulation functions of short videos. Additionally, China’s unique short video ecosystem—such as the widespread adoption and functional features of platforms like Douyin and Kuaishou—may also shape users’ usage patterns and psychological experiences. Therefore, the generalizability of these findings to other cultural contexts requires caution. Future research should conduct cross-cultural comparative studies across diverse cultural backgrounds to examine the universality and cultural specificity of the findings reported here.

## 5 Conclusion

This study investigated the associations among SVA, SCC, and depression in college students. The findings indicate that SVA does not directly predict depression. However, it is indirectly associated with depression through the mediating role of SCC, and this indirect association is primarily observed among female college students. Enhancing SCC may be a modifiable target for mitigating the risk of SVA-related depression, particularly among female university students.

## Data Availability

The datasets presented in this study can be found in online repositories. Datasets from this study are available in the Zenodo repository, accessible via https://doi.org/10.5281/zenodo.17272774. Further inquiries can be directed to the corresponding author.
